# Inhibition of H9N2 Virus Invasion into Dendritic Cells by the S-Layer Protein from *L. acidophilus* ATCC 4356

**DOI:** 10.3389/fcimb.2016.00137

**Published:** 2016-10-25

**Authors:** Xue Gao, Lulu Huang, Liqi Zhu, Chunxiao Mou, Qihang Hou, Qinghua Yu

**Affiliations:** College of Veterinary Medicine, Histology and Embryology, Nanjing Agricultural UniversityNanjing, China

**Keywords:** S-layer protein, dendritic cells, *L. acidophilus*, mucosal, avian influenza virus

## Abstract

Probiotics are essential for the prevention of virus invasion and the maintenance of the immune balance. However, the mechanism of competition between probiotics and virus are unknown. The objectives of this study were to isolate the surface layer (S-layer) protein from *L. acidophilus* ATCC 4356 as a new antiviral material, to evaluate the stimulatory effects of the S-layer protein on mouse dendritic cells (DCs) and to verify its ability to inhibit the invasion of H9N2 avian influenza virus (AIV) in DCs. We found that the S-layer protein induced DCs activation and up-regulated the IL-10 secretion. The invasion and replication of the H9N2 virus in mouse DCs was successfully demonstrated. However, the invasion of H9N2 virus into DCs could be inhibited by treatment with the S-layer protein prior to infection, which was verified by the reduced hemagglutinin (HA) and neuraminidase (NA) mRNA expression, and nucleoprotein (NP) protein expression in the DCs. Furthermore, treatment with the S-layer protein increases the Mx1, Isg15, and Ddx58 mRNA expressions, and remits the inflammatory process to inhibit H9N2 AIV infection. In conclusion, the S-layer protein stimulates the activation of mouse DCs, inhibits H9N2 virus invasion of DCs, and stimulates the IFN-I signaling pathway. Thus, the S-layer protein from *Lactobacillus* is a promising biological antiviral material for AIV prevention.

## Introduction

The mucosa contains a diverse microbial community called the microbiota, which included numerous viruses. However, the impact of the microbiota on viruses is unclear (Gerritsen et al., 2011; Guinane and Cotter, 2013). Dendritic cells (DCs) underneath the epithelial mucosa are important antigen presenting cells (APC) that play an important role in the immune response and the maintenance of the mucosal barrier (Foligne et al., 2007). DC-specific ICAM-3-grabbing nonintegrin (DC-SIGN) is a C-type lectin receptor (CLR) that can bind to mannose and other sugars commonly expressed on the surfaces of a range of viruses (Drickamer, 1992). DC-SIGN plays both a positive and a negative role in immune regulation (Svajger et al., 2010). On one hand, DCs can identify a variety of pathogens via DC-SIGN and activate naive T cells to induce an immune response. Additionally, DC-SIGN has been reported to recognize mannose and fucose on the surfaces of viruses such as HIV-1 and SARS and to mediate viral immune evasion by DCs (Alen et al., 2009; Hoorelbeke et al., 2011).

Avian influenza virus (AIV) circulates among wild birds and has contributed critical genetic material necessary for the generation of a pandemic influenza virus. Recent studies have found that AIV also belongs to a group of viruses that can bind to DC-SIGN and efficiently replicate in human DCs (Londrigan et al., 2012; Short et al., 2012). After phagocytosis of AIV in human DCs, virus particles were directly passed to other susceptible cells in lymphoid tissues (Wang et al., 2008). The immune evasion of AIV by the DC-SIGN pathway makes it difficult to prevent viruses from infecting DCs and being transmitting to other susceptible cells. The H9N2 subtype of the influenza virus is widespread in birds in Asia and the Middle East (Alexander, 2007). However, the relationship between the H9N2 virus and mouse DCs is still unclear.

Previous studies had demonstrated that *Lactobacillus*, which is present in the intestinal tract, had an antagonistic effect on various intestinal pathogenic bacteria (Jankowska et al., 2008; Martinez et al., 2015). *Lactobacillus* also stimulated DCs activation, increased IL-12 and IL-18 secretion and improved the immune response (Mohamadzadeh et al., 2005). Recent studies found that chickens fed with *Lactobacillus* could effectively prevent the replication of the influenza virus in the respiratory tract, the digestive tract and other mucosal sites (Youn et al., 2012). The S-layer protein is a crystalline array of proteinaceous subunits found in the outermost component of the cell wall in several *Lactobacillus* species (Eslami et al., 2013). The S-layer protein has antagonistic activity against enteropathogenic bacteria (Li et al., 2011a). Further studies have found that the S-layer protein could bind to the DC-SIGN receptor to regulate DCs maturation and differentiation (Konstantinov et al., 2008). The S-layer protein also inhibited JUNV invasion into 3T3 cells, which overexpress DC-SIGN, in the early stages of viral infection (Martinez et al., 2012).

In previous studies, it has been confirmed that the H9N2 virus had been transmitted from poultry to mammalian species, including humans and pigs, thereby causing a serious public health threat (Peiris et al., 1999). It was also reported that DC-SIGN is a cell-surface adhesion factor that enhances viral entry of several virus families (da Silva et al., 2011). Therefore, we explored whether the invasion of the H9N2 virus in mouse DCs could be prevented by inhibiting the binding of AIV to DC-SIGN. This strategy may prevent AIV invasion in mucosal sites and could control the spread of avian influenza. The aim of the study was to determine whether the S-layer protein from *L. acidophilus* ATCC 4356 could be used as a new biological antiviral material to compete with the H9N2 virus for binding to DC-SIGN and thus prevent the H9N2 virus from using the DC-SIGN pathway to induce invasion into DCs.

## Materials and methods

### Reagents and antibodies

RPMI 1640 medium, streptomycin, and penicillin were purchased from Invitrogen (Grand Island, NY, USA). Fetal bovine serum (FBS) was purchased from Hyclone (Thermo, Melbourne, Australia). Recombinant GM-CSF and IL-4 were purchased from Peprotech (Rocky Hill, NJ, USA). LPS (from *Escherichia coli* 026:B6) was obtained from Sigma-Aldrich (St Louis, MO, USA). The fluorescent-labeled anti-mouse CD40-PE, CD80-FITC, and CD86-PE mAbs were purchased from eBioscience (San Diego, USA). The anti-CD11c antibody and anti-influenza virus nucleoprotein antibody (FITC-conjugated) were purchased from Abcam (MA, USA).

### Viruses and animals

The influenza virus (A/Duck/NanJing/01/1000 [H9N2]) was generously supplied by the Jiangsu Academy of Agricultural Sciences (Nanjing China) (Qin et al., 2015). C57BL/6 mice (4 weeks old, specific-pathogen-free [SPF]) were purchased from the Animal Research Centre of Yangzhou University. The animal studies were approved by the Institutional Animal Care and Use Committee (IACUC) of Nanjing Agricultural University, and the National Institutes of Health guidelines for the performance of animal experiments were followed.

### Isolation and culture of bone marrow DCs

As previously described, DCs were generated from bone marrow progenitor cells with some modifications. Briefly, bone marrow was extracted from the femurs and tibias of C57BL/6 mice and treated with red blood cell lysis buffer. Then, the cells were suspended in complete medium (RPMI 1640 supplemented with 10% heat-inactivated FBS, 1% streptomycin and penicillin, and 10 ng/ml of GM-CSF and IL-4) and plated at a density at 1 × 10^6^ cells/ml in 6-well plates. After approximately 60 h of culture, the medium was gently discarded to remove non-adherent granulocytes. On day 6, the clusters were harvested and subcultured overnight to remove adherent cells. Non-adherent cells were collected on day 7 and used in subsequent studies.

### Bacterial strains and S-layer protein isolation

S-layer proteins were obtained from *L. acidophilus* ATCC 4356 as previously reported (Boot et al., 1993). Briefly, *L. acidophilus* ATCC 4356 was cultivated in MRS medium at 37°C until the logarithmic phase. The bacteria were resuspended with 4 M guanidine hydrochloride (GuHCl) for 1 h. After centrifugation (14,000 rpm, 0.5 h), the supernatant was dialyzed against distilled water overnight at 4°C and suspended in sterile PBS (0.01 M, pH 7.4). The S-layer protein was further purified by chromatography on an anion-exchange column (DE52; Whatman, Kent, UK) and stored at −70°C (Li et al., 2011b).

Cell Counting Kit-8 (CCK-8) is a nontoxic, highly sensitive colorimetric assay for the determination of cell viability in cell cytotoxicity assays. The cytotoxicity of the S-layer protein for mouse DCs was measured using the CCK-8 assay.

### Activation and adhesion of the S-layer protein to DCs

DCs grown in 12-well plates (10^6^ cells per well) were incubated with the S-layer protein at a concentration of 400 μg/ml. Untreated DCs were used as the negative control and DCs treated with LPS were used as the positive control. After 24 h of treatment, the supernatant was collected, and the DCs were washed three times with PBS. The expression of maturation markers on the DCs surface of DCs was evaluated by flow cytometry. The IL-10 and TFN-α mRNA expression levels in the supernatant were evaluated by qRT-PCR. Binding of the S-layer protein to mouse DCs was detected by immunofluorescence assay.

### Infection of DCs with the H9N2 virus

H9N2 virus (10^6^ EID_50_) was incubated with DCs grown in 12-well plates (10^6^ cells per well) at 4°C with 1 μg/ml TPCK-trypsin. After 1 h incubation, the medium was removed and fresh medium (RPMI 1640 supplemented with 2% heat-inactivated FBS, 1% streptomycin and penicillin, 10 ng/ml GM-CSF, and 1 μg/ml TPCK-trypsin) was added. DCs were collected after 1 h and evaluated by flow cytometry. Alternatively, DCs were collected at 1, 6, 12, and 24 h for qRT-PCR. The adhesion of the H9N2 virus to mouse DCs was detected by immunofluorescence assay.

### Inhibition of H9N2 viral infection of DCs

DCs grown in 12-well plates (10^6^ cells per well) were treated with different concentrations of the S-layer protein for 1 h. After removal of the culture media, the DCs were infected with the H9N2 virus in the presence of the S-layer protein. DCs were collected at 1 h post infection and then analyzed by FACS. The S-layer protein (400 μg/ml) was used for the following inhibition experiments. Additionally, DCs infected with H9N2 with or without S-layer protein treatment (400 μg/ml) were collected 1 h post-infection for qRT-PCR.

### Immunofluorescence assays

DCs were grown on glass coverslips for 4 h. Then, the cells were infected with the DyLight 488-H9N2 virus (green) for 1 h. The cells were rinsed three times with PBS, fixed with 4% formaldehyde, and permeabilized with 0.2% Triton X-100. After staining with CD11c (red) and DAPI (blue), the DCs were observed by confocal microscopy.

To verify the adhesion of the S-layer protein to the mouse DCs, the DCs were grown on glass coverslips for 4 h and cocultured with the Dylight 594-labeled S-layer protein for 1 h. Then, the cells were then rinsed three times with PBS, fixed with 4% formaldehyde, and permeabilized with 0.2% Triton X-100. The cells were cultured with an anti-DC-SIGN antibody (Abcam), incubated with the Alexa Fluor 488-conjugated secondary antibody, and observed by confocal microscopy.

To detect the adhesion of the H9N2 virus to DC-SIGN, the H9N2 virus was incubated with DCs for 1 h. Then the cells were then rinsed three times with PBS, fixed with 4% formaldehyde, and permeabilized with 0.2% Triton X-100. The cells were cultured with anti-DC-SIGN antibody (Abcam) and HA antibody, incubated with a secondary antibody, and observed by confocal microscopy.

### Quantitative RT-PCR

Infected DCs were harvested from the different treatments. Total RNA was extracted from the DCs using RNAios Plus (Takara, Dalian, China). Reverse transcription of the RNA was performed with the designed primers (Table 1), which amplified fragments of the target genes. In this experiment, 2 μl of template RNA was subjected to a real-time PCR reaction with a final volume of 20 μl using the Taq-Man PCR Master Mix (Takara, Dalian, China). The thermal cycling conditions were 5 min at 95°C, followed by 40 cycles of 15 s at 95°C and 34 s at 60°C using the Applied Biosystems 7500 real-time PCR system. The ^−ΔΔ^CT method was used for the relative virus quantification.

**Table 1:** **Primer sequences used for qRT-PCR**.

**Target genes**	**Primer sequences**
HA	Forward: 5′-AGACCATCGGCTGTTAATGG-3′ Reverse: 5′-TTGTGTATTGGGCGTCTTGA-3′
NA	Forward: 5′-TTCAGGCAGAATGAATGCAG-3′ Reverse: 5′-TGCGGAAAGCCTAATTGAGT-3′
PB1	Forward: 5′-AGCGGGTATGCACAAACAGA-3′ Reverse: 5′-ATAAGTCTGGCGACCTTGGG-3′
NP	Forward: 5′-GAAATCCTGGGAATGCTGAA-3′ Reverse: 5′-AACACCTGGCTGTTTTGGAG-3′
IL-10	Forward: 5′-GCCCAGAAATCAAGGAGCAT-3′ Reverse: 5′-TGTAGACACCTTGGTCTTGGAG-3′
TNF-α	Forward: 5′-CTTCTGTCTACTGAACTTCGGG-3′ Reverse: 5′-CAGGCTTGTCACTCGAATTTTG-3′
Isg15	Forward: 5′- GAGAGGCAGCGAACTCATCT -3′ Reverse: 5′- CTTCAGCTCTGACACCGACA -3′
Mx1	Forward: 5′-GGGGCTTTAGGCCATACTCC-3′ Reverse: 5′-TACAAAGGGCTTGCTTGCTT-3′
DDX58	Forward: 5′-AAGGAAAACTGGCCAAAGGT-3′ Reverse: 5′-TGGTTTCAATGGGCTGTGTA-3′

### Flow cytometry

Flow cytometry was used to analyse the presence of the virus in DCs and the maturation of DCs following infection with H9N2 virus. DCs were harvested after infection with the DyLight 488-H9N2 virus for 1 h and were evaluated by FACS. Furthermore, DCs infected with the H9N2 virus were collected 24 h post-infection. After washing with PBS three times, the DCs were incubated with the fluorescent-labeled anti-mouse CD86-PE, CD80-FITC, and CD40-PE mAbs for 30 min at 4°C. The cells were washed three times, suspended in 300 μl of PBS and analyzed by FACS. The Mean Fluorescence Intensity (MFI) values were calculated.

### Statistical analysis

The data were expressed as the mean ± standard error of the mean. For comparison between two groups, the data were analyzed using Student's *t*-test. For multiple groups, one-way analysis of variance (ANOVA) was used. Differences were considered statistically significant at *P* < 0.05.

## Results

### Isolation of the S-layer protein and its cytotoxic effect on DCs

**Figure 1:**
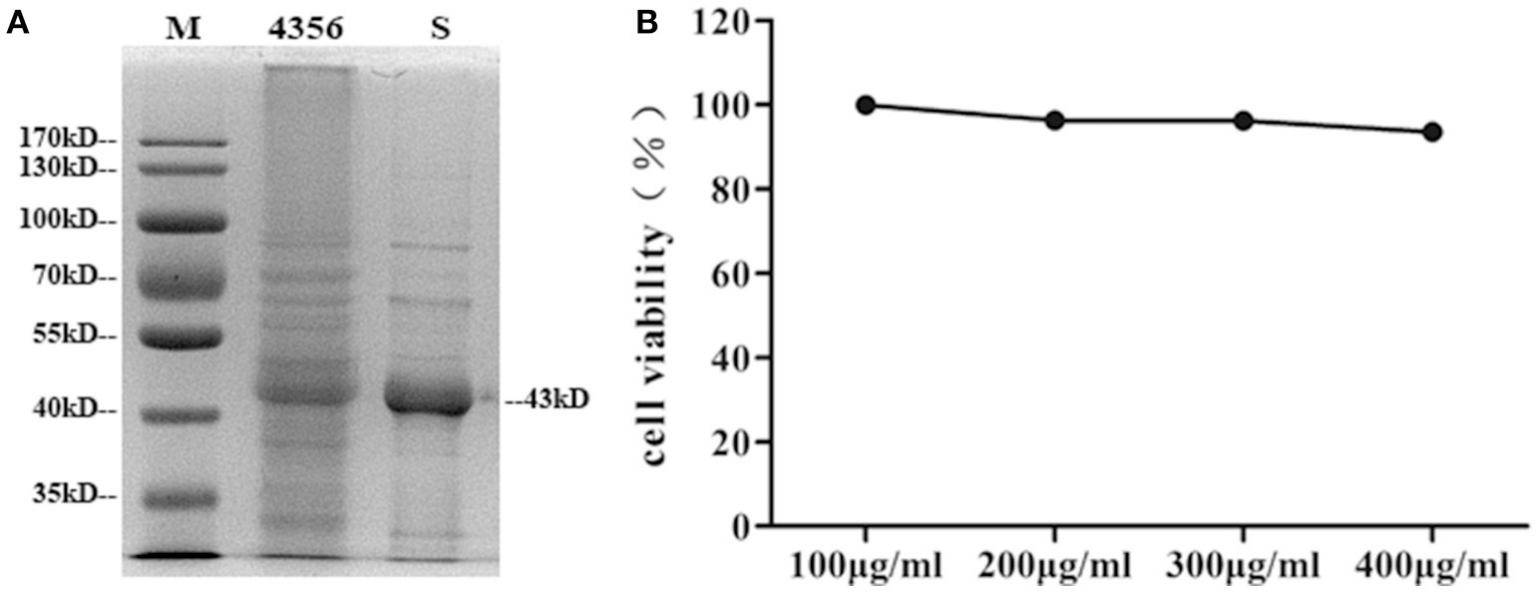
**Analysis of SDS-PAGE and the CCK-8 assay**. **(A)** The S-layer protein of *L*. *acidophilus* ATCC 4356 was extracted by GuHCl. The whole proteins of *L*. *acidophilus* ATCC 4356 and the extracted S-layer protein were analyzed by SDS-PAGE. (M, molecular weight marker. 4356, the whole proteins of *L*. *acidophilus* ATCC 4356. S, S-layer protein extracted from *L*. *acidophilus* ATCC 4356.) **(B)** The CCK-8 assay was used to assess the effect of different S-layer protein concentrations on DCs viability.

*L. acidophilus* ATCC 4356 was boiled and analyzed by SDS-polyacrylamide gel electrophoresis (SDS-PAGE) to confirm that the strain we used contained the S-layer protein. The 43 kDa band corresponding to the S-layer protein was visible (Boot et al., 1993; Figure 1A). Then, the S-layer protein was extracted from *L. acidophilus* ATCC 4356 with GuHCl (Figure 1A). To determine the cytotoxic effect of the S-layer protein extracted from *L. acidophilus* ATCC 4356, we used the CCK-8 assay to detect cell viability after treatment with different concentrations of the S-layer protein (Figure 1B). We found that none of the tested concentrations exerted a cytotoxic effect on DCs.

### The stimulatory effect of the S-layer protein on DCs

**Figure 2:**
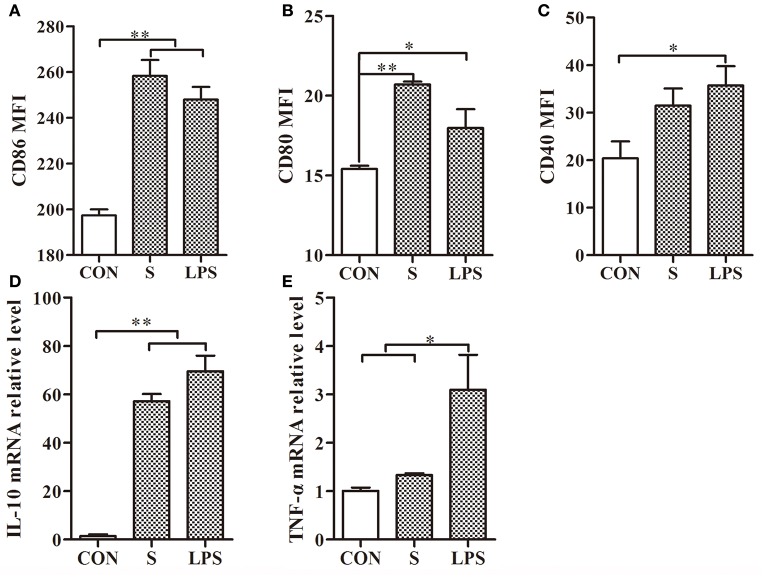
**S-layer protein activates DCs through up-regulation of maturation marker expression and cytokine production. (A–C)** Flow cytometry analysis of the maturation markers CD86, CD80, and CD40 expressed on the surface of DCs treated with the S-layer protein (400 μg/ml) is shown. **(D,E)** The relative mRNA levels of IL-10 and TNF-α in DCs incubated with the S-layer protein (400 μg/ml) for 24 h were analyzed by qRT-PCR. CON, relative mRNA level in untreated DCs in these experiments; LPS, relative mRNA level in DCs treated with LPS (10 ng/ml); ^*^P < 0.05; ^**^P < 0.01. Results are from three different experiments.

After treatment of mouse DCs with the S-layer protein extracted from *L. acidophilus* ATCC 4356, CD86 and CD80 expressions were significantly increased but CD40 expression was not (Figures 2A–C). Furthermore, significant up-regulation was observed for CD86 and CD80, which surpassed the positive control (LPS). These observations suggested that the S-layer protein could modulate DCs maturation by activating CD86 and CD80 expression. Moreover, the results indicated that the S-layer protein could induce a very high IL-10 expression level and no significant up-regulation of TNF-α (Figures 2D,E). Thus, the S-layer protein could activate DCs maturation by up-regulating cell surface maker expression and cytokine secretion.

### Infection of DCs by the H9N2 virus

**Figure 3:**
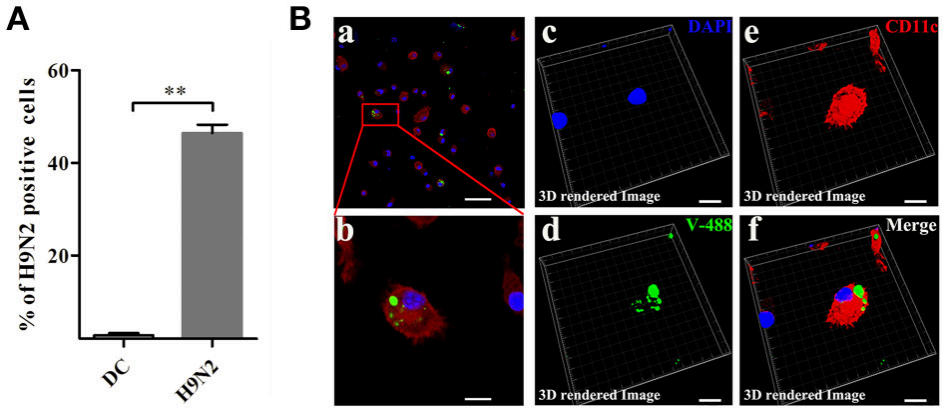
**H9N2 virus infects DCs. (A)** DCs infected with the DyLight 488-H9N2 virus (green) for 1 h were analyzed by FACS. ^**^P < 0.01. **(Ba,b)** Confocal microscopy was used to observe the invasion of DCs by the DyLight 488-H9N2 virus. **(Bc–f)** Three-dimensional rendering of the images obtained using the Imaris 7.2 software. DCs were stained with CD11c (red) and DAPI (blue). Bars: 50 μm **(Ba)**; 10 μm **(Bb–f)**.

To investigate the invasion possibility of the H9N2 virus in DCs, DCs were infected with the DyLight 488-H9N2 virus for 1 h. Untreated DCs was used as a negative control. The number of DCs infected with the DyLight 488-H9N2 virus was assessed by flow cytometry. Compared with untreated DCs, the DyLight 488-H9N2 virus was detected in DCs infected with the H9N2 virus (Figure 3A). Moreover, the DyLight 488-H9N2 virus was clearly found in the cytoplasm of DCs by confocal microscopy (Figures 3Ba,Bb) and by image analysis using the Imaris 7.2 software (Figures 3Bc–f). These results demonstrated that the H9N2 virus could invade mouse DCs.

### The effect of the H9N2 virus on mouse DCs

**Figure 4:**
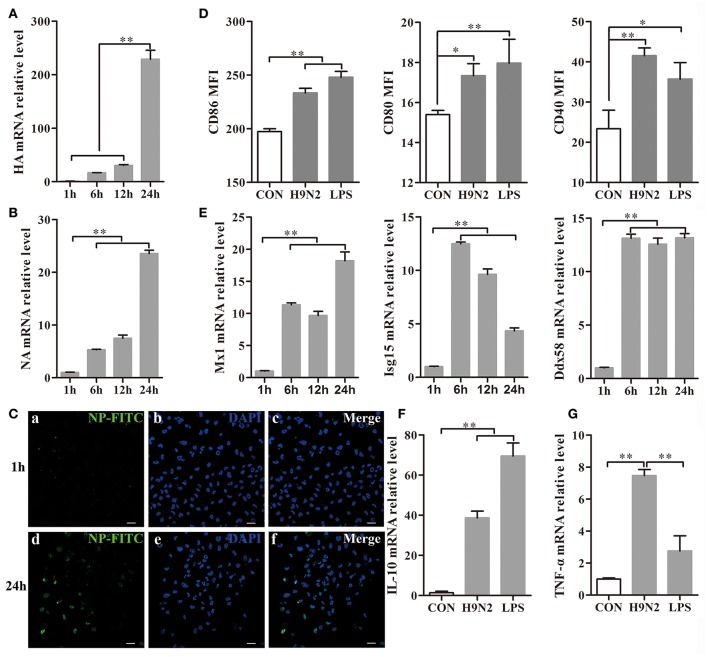
**Efficient replication of the H9N2 virus in DCs and the response of DCs treated with the H9N2 virus**. DCs infected with the H9N2 virus were harvested at different time points after infection as indicated. **(A,B)** The relative HA and NA mRNA levels were evaluated by qRT-PCR. The expression levels of these genes in DCs treated with the H9N2 virus is presented in relation to untreated DCs. **(C)** The H9N2 virus was detected in DCs by confocal microscopy at 1 h and 24 h post-infection. Bars: 20 μm **(D)** Then, CD86, CD80, and CD40 expression on the surface of treated and untreated DCs was analyzed at 24 h post-infection by FACS. **(E)** The relative mRNA levels of Mx1, Isg15, and Ddx58 were evaluated by qRT-PCR. The expression levels of these genes in DCs treated with the H9N2 virus are presented in relation to untreated DCs. **(F,G)** The gene transcription levels of IL-10 and TNF-α in DCs were analyzed by qRT-PCR 24 h post-infection. ^*^P < 0.05; ^**^P < 0.01. The results are from three different experiments.

DCs were collected 1, 6, 12, and 24 h after H9N2 viral infection. During the infection, the hemagglutinin (HA) and neuraminidase (NA) mRNA expression levels were markedly increased in the DCs (Figures 4A,B), which implied that the H9N2 virus was able to productively replicate in mouse DCs. The replication process was also further confirmed by the detection of FITC-labeled NP in DCs infected with the H9N2 virus for 24 h in the immunofluorescence assay (Figure 4C).

After infection with the H9N2 virus for 24 h, the CD86, CD80, and CD40 expression levels in the DCs were significantly increased compared to the untreated group (Figure 4D). Mx1, Isg15, and Ddx58 are interferon-stimulated genes (ISGs) triggered by the type I interferon (IFN) system induced by many viruses (Schoggins and Rice, 2011). The Mx1, Isg15, and Ddx58 mRNA expressions levels were significantly increased after virus infection (Figure 4E). The Mx1 expression reached its peak at 24 h post-infection, whereas Isg15 expression declined from the peak at 6 h. Ddx58 mRNA expression was maintained at a high level from 6 h to 24 h. These results showed that the H9N2 virus was able to replicate productively and induce cell responses in DCs. The relative IL-10 and TNF-α mRNA expression levels produced by the DCs were significantly up-regulated 24 h post-infection with the H9N2 virus (Figures 4F,G). These findings indicated that the H9N2 virus not only was able to infect DCs but also replicated efficiently in DCs and induced a strong response of DCs.

### Antagonistic effect of the S-layer protein against the H9N2 virus

**Figure 5:**
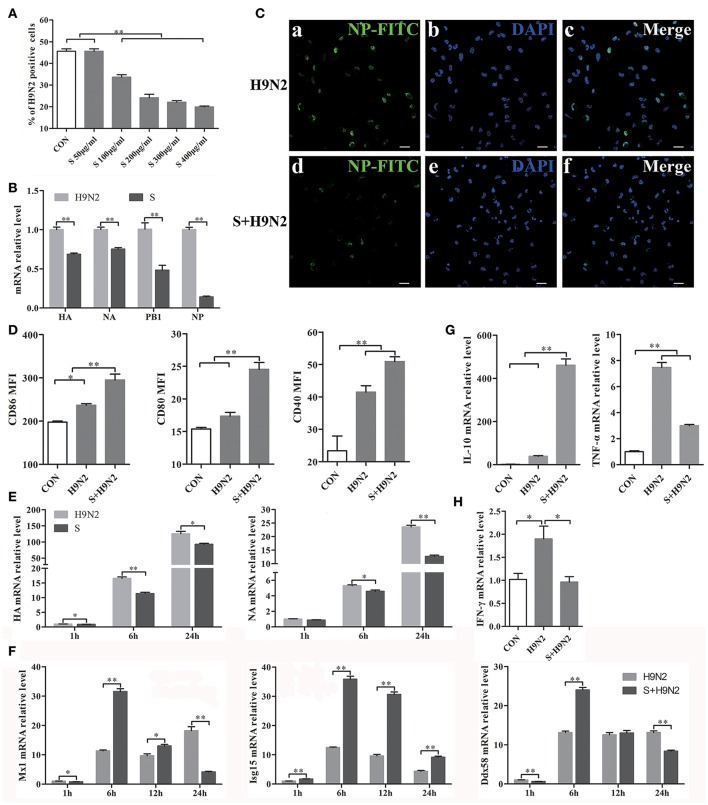
**S-layer protein inhibits H9N2 virus invasion of DCs and leading to a series of changes in the DCs. (A)** Flow cytometry was used to detect DyLight 488-H9N2 virus invading into DCs which incubated with different concentrations of S-layer protein before infected with H9N2 virus. The number of DCs infected with the DyLight 488-H9N2 virus is shown. **(B)** The mRNA relative expression levels of HA, NA, PB1, and NP 1 h after the infection of the DCs by H9N2 virus was analyzed by qRT-PCR. **(C)** H9N2 virus in the DCs treated with and untreated with S-layer protein was detected by confocal microscopy 24 h post-infection. Bars: 20 μm. **(D)** CD86, CD80, and CD40 expression on the surface of DCs under different treatments was analyzed by FACS after 24 h. **(E,F)** DCs infected with the H9N2 virus after incubation with the S-layer protein at different time points were collected for comparison of the mRNA expression levels of HA, NA, and some ISGs (Mx1, Isg15, and Ddx58) to DCs untreated with the S-layer protein. **(G)** The relative mRNA expression levels of IL-10 and TNF-α24 h post-infection were evaluated by qRT-PCR. **(H)** The gene transcription levels of IFN-γ in DCs treated with the S-layer protein and untreated were evaluated by qRT-PCR 1 h post-infection with the H9N2 virus. ^*^P < 0.05; ^**^P < 0.01. The results are from three different experiments.

DCs were assessed by flow cytometry after infection with the DyLight 488-H9N2 virus for 1 h. The results showed that the number of DCs infected with the H9N2 virus was significantly reduced in the presence of the S-layer protein beginning at a concentration of 100 μg/ml. Furthermore, the inhibitory effect was more efficient with the increasing concentrations of the S-layer protein (Figure 5A), suggesting that the S-layer protein had a dose-dependent antagonistic effect against H9N2 virus infection of DCs. The relative HA, NA, PB1, and NP mRNA expression levels were significantly reduced 1 h post-infection, which indicated that DCs treated with the S-layer protein became less susceptible to H9N2 virus infection than untreated DCs (Figure 5B). Additionally, the immunofluorescence assay results showed a similar antiviral tendency against H9N2 AIV invasion (Figure 5C). The expression of CD86, CD80, and CD40 in DCs cocultured with the S-layer protein and the H9N2 virus were significantly increased compared to DCs untreated or treated with the H9N2 virus (Figure 5D). We also found that the S-layer protein not only inhibited the virus invasion after 1 h of incubation but also suppressed the replication of the H9N2 virus in DCs up to 24 h post-infection. HA and NA mRNA expression in DCs treated with the S-layer protein appeared to be significantly reduced compared to the untreated DCs 1, 6, and 24 post-infection with the H9N2 virus (Figure 5E). The ISGs expression levels (Mx1, Isg15, and Ddx58) were significantly increased at 6 h (Figure 5F). Moreover, when the DCs were treated with the S-layer proteins, IL-10 mRNA expression was up-regulated and TNF-α mRNA expression was decreased (Figure 5G). Finally, the IFN-γ gene transcription level in DCs treated with the S-layer protein was reduced (Figure 5H). All of these results indicated the S-layer protein prevented H9N2 virus invasion of mouse DCs.

## Discussion

DCs respond dynamically to microbes in their environment by undergoing changes that enhance their capacity to capture and process antigens, load peptides onto MHC molecules and transport them to the cell surface (Banchereau et al., 2000). In our study, 4 M GuHCl was used to extract the S-layer protein from *L. acidophilus* ATCC 4356. One dominant 43 kDa band appeared when the extract was analyzed by SDS-PAGE. To test the cytotoxicity of the S-layer protein for cells, we evaluated cell viability using the CCK-8 assay. The results showed that the S-layer protein was a highly stable protein with no toxicity for DCs, which is essential for biological antiviral material.

Co-stimulatory molecules have been used to measure effects on DCs (Acevedo et al., 2013). During immune processes, cytokines play an important role in inducing a variety of biological effects on different cell types. Our data confirmed that the isolated S-layer protein was involved in regulating mouse DCs maturation and stimulating cytokine release. This finding indicates that the S-layer protein participates in the immune response and promotes DCs maturation, leading to the enhancement of its effect on antigen presentation and T cell activation. At the same time, the S-layer protein induces IL-10 production, which inhibits the inflammatory response and promotes the differentiation of naive CD4^+^ T cells into Th2 cells (Seder et al., 1992). These results indicate that the S-layer protein plays a role in regulating inflammation. The co-localization of the S-layer protein with DC-SIGN was also confirmed by immunofluorescence (Figure S1).

H9N2 viral infection may be asymptomatic, which allows the virus to remain unrecognized by surveillance systems. In this study, the flow cytometry and confocal microscopy data demonstrated that the H9N2 virus not only infected DCs but also replicated in DCs over a period of time. This result was confirmed by an increase in the relative HA and NA mRNA levels in DCs. Moreover, the H9N2 virus could invade DCs through attachment to DC-SIGN (Figure S2). H9N2 virus infection also significantly up-regulated the transcriptional expression of ISGs. We confirmed that the transcriptional expression of Mx1 and Ddx58 was increased over time during infection. Infection with the H9N2 virus leads to an increase in the expression of the pro-inflammatory cytokines TNF-α. However, the relative mRNA level of IL-10, which inhibits inflammation, also exhibited obvious up-regulation. Viruses can stimulate DCs, which efficiently prime and cross-prime antigen-specific T cells and then activate T cells to initiate the immune response (Manches et al., 2014). Stimulation by the H9N2 virus led to DCs maturation by enhancing their presentation ability via increasing CD86, CD80, and CD40 expression. These results demonstrated that the H9N2 virus could infect DCs and efficiently replicate in the cells. Furthermore, infection and replication both led to a series of changes associated with the initiation of inflammation.

AIV invasion is mediated by the SA α2,3-Gal receptors on epithelial cells in mucosal tissue (Shen et al., 2011). Additionally, the uptake and presentation of DCs in the lamina propria under the mucosal epithelium is a prerequisite for initiation of the immune response. By preventing the interaction between AIV and DCs at mucosal sites, inhibition of the spread of avian influenza could be achieved. To determine whether the S-layer protein could inhibit H9N2 infection of DCs, we analyzed DCs treated with the S-layer protein prior to infection with the H9N2 virus. The results of flow cytometry, qRT-PCR, and confocal microscopy results showed that the S-layer protein of *L. acidophilus* ATCC 4356 had an inhibitory effect on H9N2 virus infection. Additionally, secretion of the anti-inflammatory cytokine IL-10 by DCs treated with the S-layer protein was significantly higher than secretion from DCs infected with only the H9N2 virus, whereas the pro-inflammatory cytokine TNF-α exhibited an opposing trend. Inflammation can promote the recruitment of immune cells, but uncontrolled and exacerbated inflammation, as observed in many human AIV cases, is associated with systemic edema and extensive tissue damage (Wang et al., 2016). In our study, we found that S-layer protein treatment increased IL-10 expression, which aided in the control of the exacerbated inflammation, caused by AIV infection. The increased IL-10 level is also consistent with the TNF-α reduction, because these cytokines function together to control inflammation. Considering the uncontrolled and exacerbated inflammation caused by AIV, a suitable reduction in inflammation may be helpful for the prevention of AIV infection. These data indicated that S-layer protein might suppress the inflammatory response with the inhibition of H9N2 virus.

Previous reports showed that infection with the H9N2 virus could induce a strong IFN response (Grouard et al., 1997; Sutejo et al., 2012; Westenius et al., 2014). When H9N2 viral infection was inhibited by the S-layer protein, the relative IFN-γ mRNA level was also decreased. This result confirms that the S-layer protein acts as an inhibitor of H9N2 viral infection of DCs. Furthermore, the changes in the HA, NA, and ISGs gene transcription levels indicated that the S-layer protein could maintain the inhibition for up to 24 h post-infection with the H9N2 virus. This ability makes the S-layer protein an antagonist in the fight against viral invasion.

**Figure 6:**
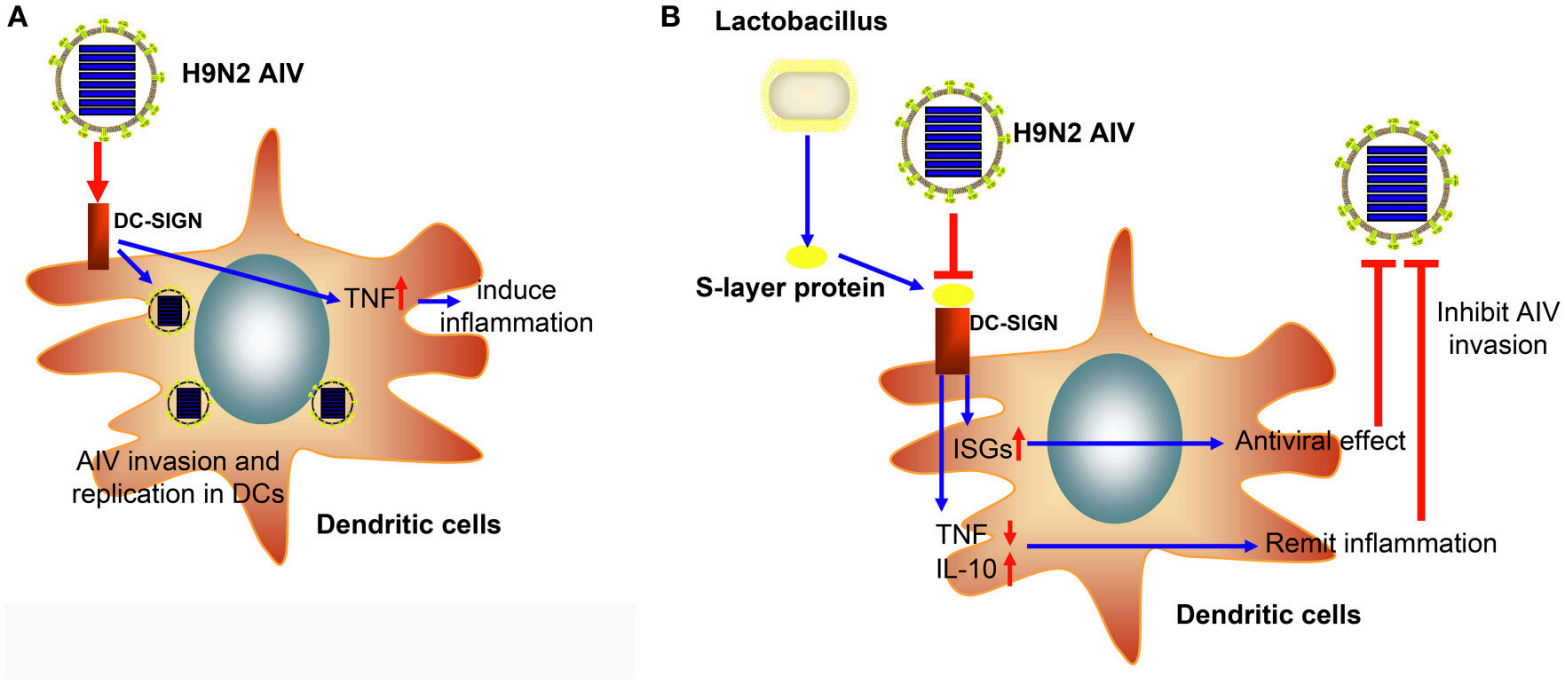
**The schematic diagram of the inhibitory effect of the S-layer protein against H9N2 AIV. (A)** The H9N2 AIV could invade DCs through DC-SIGN. **(B)** The S-layer protein isolated from *Lactobacillus* could compete with H9N2 AIV for binding to DC-SIGN and inhibit AIV invasion into DCs.

In conclusion, we successfully demonstrated that the H9N2 virus could invade and replicate in mouse DCs. Moreover, the S-layer protein of *L. acidophilus* ATCC 4356 could suppress H9N2 viral infection in DCs, stimulate the IFN-I signal pathway, and suppress the inflammatory process (Figure 6). This study demonstrates that the S-layer protein is a new biological material that is effective at inhibiting H9N2 AIV invasion in DCs. Our results provide a new method for the prevention of AIV epidemic disease. Furthermore, this study opens the possibility of using a natural characteristic of bacteria, which live together with viruses in the mucosa, to interrupt or inhibit the viral invasion process.

## Author contributions

XG: study conception and design, performance of the experiments, data analysis and interpretation, manuscript writing; LH and LZ: performance of the experiments, data analysis; CM and QH: cell isolation and culture, data analysis and interpretation; QY: study conception and design, financial support, administrative support, data analysis and interpretation, manuscript writing, final approval of the manuscript.

### Conflict of interest statement

The authors declare that the research was conducted in the absence of any commercial or financial relationships that could be construed as a potential conflict of interest.

## Supplementary material

Figure S1 **The adhesion of the S-layer protein to DC-SIGN**. DCs were cocultured with the S-layer protein for 1 h and analyzed by confocal microscopy. Bars: 20 μm.

Figure S2 **The adhesion of the H9N2 virus to DC-SIGN**. DCs were cocultured with H9N2 virus for 1 h and analyzed by confocal microscopy. Bars: 20 μm.
