# Supplementary figures and images for: Inhibition of H9N2 Virus Invasion into Dendritic Cells by the S-Layer Protein from L. acidophilus ATCC 4356
Source: Front Cell Infect Microbiol. 2016 Oct 25;6:137. doi: 10.3389/fcimb.2016.00137 (PMC5078685; doi:10.3389/fcimb.2016.00137)

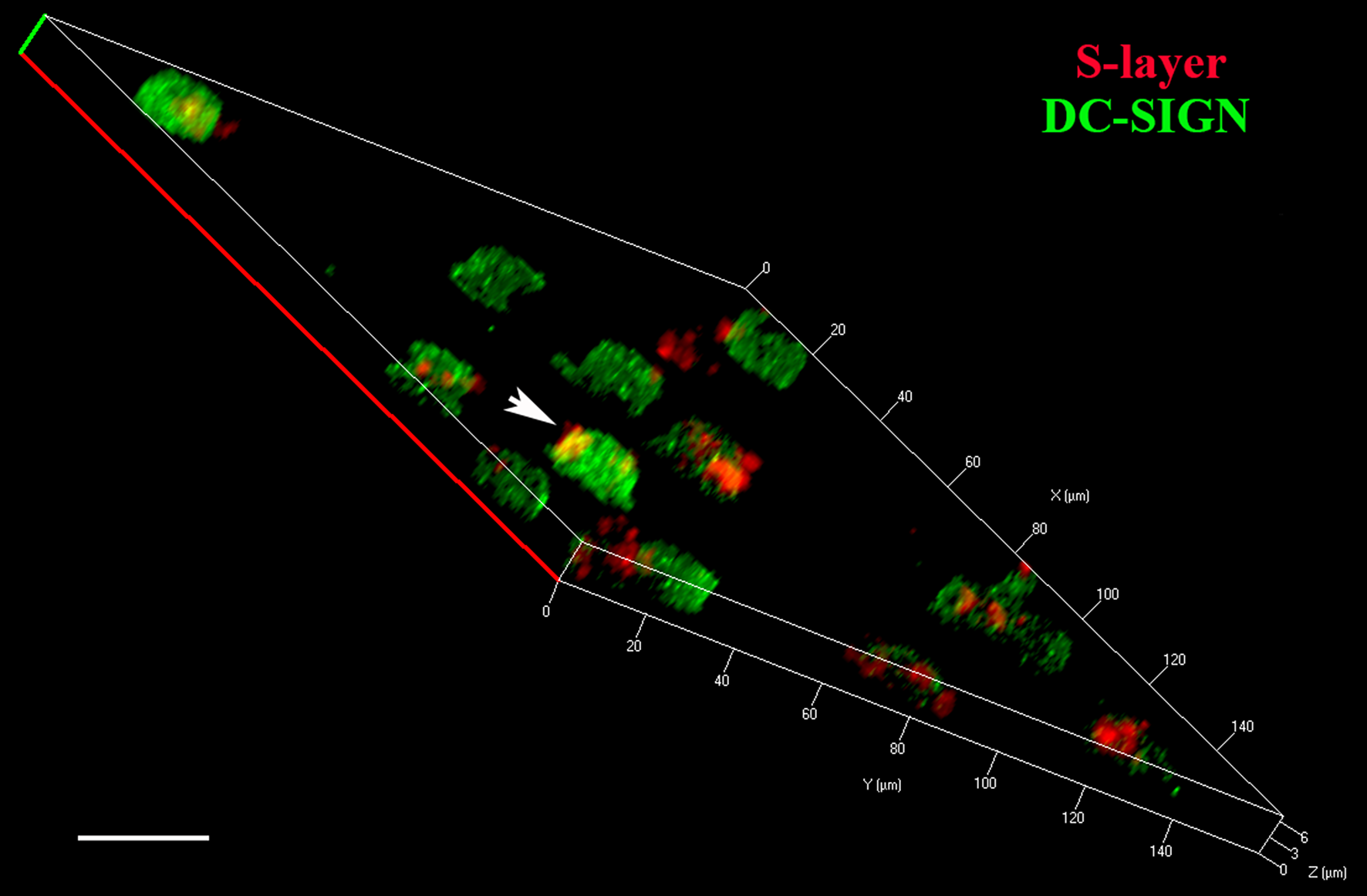

Supplement: Figure S1 — The adhesion of the S-layer protein to DC-SIGN. DCs were cocultured with the S-layer protein for 1 h and analyzed by confocal microscopy. Bars: 20 μm. [file Image1.TIF]

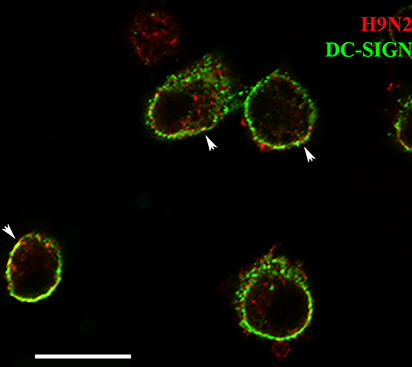

Supplement: Figure S2 — The adhesion of the H9N2 virus to DC-SIGN. DCs were cocultured with H9N2 virus for 1 h and analyzed by confocal microscopy. Bars: 20 μm. [file Image2.TIF]
